# Promoting the Concept Healthy Ageing for Use in Gerontological Health and Social Care Policy and Practice

**DOI:** 10.1111/jocn.17558

**Published:** 2024-11-18

**Authors:** C. Donnellan

**Affiliations:** ^1^ School of Nursing and Midwifery Edith Cowan University Joondalup Australia; ^2^ School of Nursing and Midwifery, Faculty of Health Sciences Trinity College Dublin Dublin Ireland

**Keywords:** ageing metrics, ageing theoretical frameworks, gerontology policy, health professionals, healthy ageing, successful ageing

## Abstract

**Aims:**

The concept of healthy or successful ageing dates back to the 1960s, where its goal is more realistic in today's ageing society as a result of effective interventions to control and reduce disability and health risks. The aim of this paper is to outline the importance of defining ageing, the semantics and indicators used, and to identify common challenges for health professionals' understanding and application of a healthy ageing approach in their everyday clinical practice.

**Design and Methods:**

This discursive paper demonstrates how realistic ageing indicators are for highlighting the variation and impact of challenges associated with ageing. It examines the proportion of older adults requiring aged‐care services and allocation of resources, focusing on health maintenance and secondary ageing prevention.

**Results:**

Indicators of ageing commonly used in clinical healthcare settings are reviewed, and their appropriateness for determining functional and intrinsic capacity of older adults. Other indicators are introduced, i.e., the Healthy Life Expectancy (HLE), Disability Free Life Expectancy (DFLE), the Human Development Index (HDI), and the Active Ageing Index (AAI), for enhancing and promoting a healthy ageing model of healthcare. Healthy ageing models of health and social care are also considered.

**Conclusions:**

Outlining evidence on healthy ageing may facilitate health professionals to address realistic challenges regarding age‐related health and social care provision, using a personalised approach for every older adult as opposed to cutting off chronological age parameters.

**Relevance to Clinical Practice:**

Increasing health professionals' focus on healthy ageing will maintain good health in at least 80% of the ageing population for longer.

**Patient or Public Contribution:**

No patient or public contribution.

**Summary**

- What does this paper contribute to the wider global clinical community?
  ○ Healthy ageing is a relatively recent field of interest in gerontological care for idenitfying the challenges it poses for the design of health and social policy and practice.
  ○ Recognising what ageing indicators are realistic for identifying functional and intrinsic capacity associated with ageing.
  ○ The importance of encourageing healthy ageing in supporting the demands of a growing ageing population.
  ○ Shifting health professionals' mindset in relation to ageing from a pathology‐orientated medical model to using a holistic healthy ageing framework that supports maintenance of high physical and cognitive function and sustained engagement in social and productive activities.

## 1 Introduction

Population ageing is the process by which older adults become a proportionally larger share of the total population and this was one of the most distinctive demographic events of the 20th century with fertility decline been the primary determinant. The global population aged 65 years and over is set to increase from 780 million in 2022 to 1.6 billion in 2050 (United Nations 2022). Life expectancy figures have increased more than 6 years between 2000 and 2019 from 66.8 years in 2000 to 73.4 years in 2019 and healthy life expectancy figures by 8% from 58.3 in 2000 to 63.7, in 2019 (World Health Organsiation 2020). Many current social, psychological, and biological approaches for older adults use chronological indicators to base their interventions on and assume that people become old at age 65 years although 65‐year‐old adults today live longer than their previous historic ancestors. Longevity figures globally not only shows that more individuals are surviving to old age but once there, they tend to live longer, meaning there are expected relative gains in life expectancy. Given the dramatic rise and demographical change of older adults living longer within communities, there has never been a greater need for health professionals and policy makers to be at the forefront for this ongoing change challenging health and social care provision.

However, during the recent global pandemic of COVID 19 that affected most countries globally from March 2020, older populations were referred to as vulnerable, disabled and if over a given chronological age i.e., 70 years and above, they were deemed incapable of making their own health, safety, and welfare decisions. This was largely echoed by health professionals and health and social care management in most instances and additionally, individuals beyond this age parameter were also referred to as elderly and incapacitated in media coverage regardless of their physical health and actual abilities in living their everyday lives prior to the COVID 19 pandemic. This prompted the aim for this special article to outline and summarise the evidence regarding the concept healthy ageing in facilitating health professionals and policy makers address some of the challenges for age‐related health and social care and provide appropriate management in accordance with older adults' actual health needs and not according to chronological age estimates. It will also identify how realistic our ageing indicators are, and how the promotion of healthy and successful ageing would have greater impact in supporting the ongoing demands of an ageing population.

## 2 Defining Ageing

In clinical settings, health professionals tend to refer to many older adults as aged or requiring aged care in the provision of health and social care. Historically the common terms used to represent older adults were elderly and/or frail individuals despite their functional and intrinsic capacity. There can be biased mindsets in the provision of health and social care in presuming all older adults will have a gradual decrease in physical and mental capabilities requiring interventions. There is a public perception especially among health professionals, that all older adults are at a growing risk of disease and ultimately death once they reach certain chronological ages and this can be as young as 50–55 years in some regions e.g., low‐ and middle‐income countries. Albeit these changes are seen in some older adults as they chronologically age but given the diversity across individuals, these are not consistent changes for all adults as they progress into older decades of the life course.

### 2.1 Semantics Used to Define Ageing

There have been various terms or semantics associated with ageing that reflect the concept positively. The substantial increases in life expectancy at birth achieved over the previous century, combined with medical advances, escalating health and social care costs, and higher expectations for older age, have led to international interest in how to promote a healthier old age and how to age successfully (Bowling and Dieppe 2005). The concept of successful ageing dates back to the 1960s (Havighurst 1963) and included the elements life satisfaction and active engagement with life. The goal of successful ageing is now more realistic in today's ageing society because of more effective interventions to control and reduce disability and health risks. It has recently been proposed as a field of interest in gerontological research and as a challenge for the design of social policy (Baltes and Baltes 1990). According to Rowe and Kahn (1997), successful ageing is multidimensional, encompassing the avoidance of disease and disability, the maintenance of high physical and cognitive function, and sustained engagement in social and productive activities (Rowe and Kahn 1997). Bowling and Dieppe (2005) outline the main theoretical approaches that define successful ageing as psychosocial and biomedical models and include additional lay definitions (Bowling and Dieppe 2005). The biomedical model focuses on the absence of disease and the maintenance of physical and mental functioning, whereas psychosocial models focus on life satisfaction, social participation and functioning, and psychological resources, including personal growth. Psychological resources are required for successful ageing, and according to Bowling and Dieppe (2005), these include a positive outlook and self‐worth, self‐efficacy, or sense of control over life, autonomy and independence, and effective coping and adaptive strategies in the face of changing circumstances. Psychosocial researchers generally agree on defining successful ageing as subjective well‐being, life satisfaction, and longevity (Freund and Riediger 2003; Jopp and Smith 2006). As a result of longevity, people are now living longer with physical impairments, whether acquired for the first time in old age or from a younger stage in life. Although different authors have defined ageing in this positive light (see Table 1), the shift has been from successful ageing to healthy or active ageing as a result of some criticism of the term successful (Katz and Calasanti 2015; Martin et al. 2015) and in line with the more recent World Health Organsiation (2015) report on ageing and health (World Health Organsiation 2015; Table 2).

**TABLE 1:** Semantics of ageing definitions.

Semantic	Definition	Author and year
Healthy	Process of developing and maintaining the functional ability that enables wellbeing in older age Intrinsic capacity and functional ability	(World Health Organisation 2015)
Successful	Avoidance of disease and disability, maintenance of high physical and cognitive function, and sustained engagement in social and productive activities	(Havighurst 1963) (Baltes and Baltes 1990) (Rowe and Kahn 1997)
Active	Process of optimising opportunities for health, participation and security in order to enhance quality of life as people age	(World Health 2002)
Positive	A diversity of positive conditions across the ageing process and during old age, such as physical fitness and health, optimal cognitive functioning, positive emotional states, and social involvement	(Fernández‐Ballesteros 2011)
Productive	An umbrella concept covering the different types of activities that people engage in older ages	(Dommaraju and Wong 2021)
Harmonious	Stresses the complementary coexistence of body and mind, harmonious family and social relationships, and a balanced outlook that appreciates both opportunities and challenges in old age	(Liang and Luo 2012)
Meaningful	Goes beyond what a person has or achieves and what a person did, or has been doing, but centres on what a person is being, or being in relation with events, people, and relationships. Conceptualised in relation to how a person makes sense of himself and his surroundings and the relationship between them	(de Medeiros and O'Neill 2020) (Lou 2022)
Ageing well	A dynamic, interactive process that creates long‐term, positive change by involving individuals in the physical, social, economic, historical, and cultural contexts of their environments.	(Hawkins 2005)

**TABLE 2:** Indicators used to classify ageing populations.

Indicator	Definition
Old‐age dependency ratio	Number of individuals aged 65 or older per 100 people of working age, defined as those aged between 20 and 64 years old
Nominal age	Nominal age is chronological age or years of living
Real age	The body's health age or age adjusted for life expectancy or changes in mortality rates
Chronological age	One's length of existence or years of living
Biological age	Age of human cells
Phenoage	Measure of biological (physiological) age calculated based on chronological age plus albumin, creatinine, glucose, CRP, % lymphocyte, mean red blood cell volume and distribution, weight, alkaline phosphatase, and White blood cell count
Third age	Aged 65 years and older in lifespan psychology
Fourth age	Aged 80 years and older in lifespan psychology
Healthy Life Expectancy (HLE)	Average number of years that a person can expect to live in “full health” by considering years lived in less than full health due to disease and/or injury
Disability Free Life Expectancy (DFLE)	Average number of years a person could expect to live without disability
Active Ageing Index (AAI)	Measures the level to which older people live independent lives, participate in paid employment and engage social activities, as well as their capacity for active ageing
Human Development Index (HDI)	A statistical composite index of life expectancy, education (mean years of schooling completed and expected years of schooling upon entering the education system), and per capita income indicators

### 2.2 Indicators Used to Classify Ageing Populations

Two commonly used indicators of ageing are the proportion of the population aged 65 years and older and the old‐age dependency ratio, i.e., the number of persons 65 years and over per one hundred persons 15–64 years. The 65 years of age cut‐off age was predominantly determined to be in keeping with the most common global perspectives on what should be retirement age (Costa 1998). Other common distinctions used are nominal and real ages. Nominal ages are chronological ages and real ages are ages adjusted for life expectancy or changes in mortality rates (Fuchs 1984). Chronological age refers to length of existence and is more commonly compared to one's biological age which is determined by the age of human cells. Some individuals have similar chronological and biological ages; however, for others, these two numbers may not be the same pending on their physiological health status. Biological age (also called PhenoAge) is considered a more superior and accurate measure for determining health status and health outcomes (Hamczyk et al. 2020), and some evidence shows it can predict hospital mortality (Ho et al. 2023). In lifespan development literature, aged 65 and older is referred to as the third age and 80 and older goes into the fourth age to be inclusive of octogenarians, nonagenarians, and centenarians (Baltes and Smith 2003). These indicators do not take into consideration the health and activity levels of individuals as they grow older.

Indicators less commonly used to categorise or classify ageing cohorts in health and social care settings are the Healthy Life Expectancy (HLE) and Disability Free Life Expectancy (DFLE) indices that illustrate length of time spent in different states of health. These are more commonly used to report global and national population statistics as part of longevity figures by the Global Health Observatory on behalf of the World Health Organisation (2015, 2020). The DFLE is the average number of years that an individual can expect to live free from a limiting persistent illness or disability in their lifetime and the HLE is the average number of years a person can expect to live in full health by considering years lived in less than full health due to disease and/or injury. The DFLE and HLE are useful figures for determining the extent of health disparities, with evidence reported of associations found between these figures and disparities in race, gender, ethnicity, education, and socioeconomic status (Galvin, Friedman, and Hébert 2021; Li et al. 2024). The least common indices considered by health and social care policies and services in relation to health and social care decision‐making relevant for older populations are those that classify ageing in the context of healthy and active ageing, such as the Active Ageing Index (AAI) (UNECE/European Commission 2018) and the Human Development Index (HDI) (United Nations Development Programme 2024).

The AAI is a tool developed to measure the untapped potential of older adults across different countries and has been used extensively within the European Union to compare the active ageing potential of member states, thereby helping tailor country‐specific policies (UNECE/European Commission 2018). It focuses on multiple dimensions that include employment, participation in society, independent, healthy, and secure living and capacity, and an enabling environment for active ageing, providing a holistic view of the ageing experience and helping identify areas for policy intervention and improvement. The index is particularly useful for policymakers, researchers, and stakeholders interested in promoting the well‐being and active participation of older individuals in society. The HDI is a composite statistic used to rank countries based on human development levels and was created to emphasise that people and their capabilities should be the ultimate criteria for assessing the development of a country, not economic growth alone (United Nations Development Programme 2024). It is inclusive of three dimensions: a long and healthy life as measured by life expectancy at birth, knowledge measured by expected years of schooling (for children of school entering age) and average years of schooling (for adults aged 25 and older), and a decent standard of living measured by gross national income per capita (United Nations Development Programme 2024). The HDI and ageing are interconnected areas of study that together provide a comprehensive understanding of how well countries are doing in promoting the well‐being of their populations, particularly older adults. The HDI provides a summary measure of human development, focusing on expanding people's choices and enhancing their capabilities. Understanding the intersection of the HDI and ageing is crucial for developing policies that promote the well‐being of older adults with a specific focus on life expectancy, education, and income security. The benefits of using HDI and AAI as indices to categorise or classify ageing populations include benchmarking and comparing holistic older adults' capacity across regions and countries and determining where improvements are needed over time. These insights can guide the creation of targeted policies to promote and raise public awareness of healthy and active ageing and the contributions of older adults to society in acknowledgement of the longevity dividend (Olshansky 2016). The term was first coined by Olshansky and colleagues to acknowledge the biological efforts for slowing down ageing and the social, economic, and health benefits that would result from such advances may be thought of as longevity dividends and pursued as the new approach to health promotion and disease prevention in the 21st century (Olshansky et al. 2007).

## 3 Acknowledging Health‐Related Challenges Associated With Ageing

Identifying realistic proportions of older adults living with health‐related challenges and conditions associated with ageing is essential for forward planning of health and social care management of resources required in response to ageing populations. It is well recognised that higher proportions of older adults are living with one or more chronic conditions supported by the global burden of disease data (Global Burden of Disease 2019 Ageing Collaborators 2022; The Global Burden of Metabolic Risk Factors for Chronic Diseases Collaboration 2014). The proportion of adults aged 65 years and older with two or less chronic conditions is 77% in the United States (Ansah and Chiu 2022) and 95% in Australia for those having at least one chronic condition (Australian Bureau of Statistics 2021). Chronic diseases most prevalent among older adults include cardiovascular disease and stroke, diabetes, arthritis, and hypertension; and other health‐related challenges for older adults' concern issues with brain and mental health to include mild cognitive impairment and dementia; and sensory deficits, including vision and hearing loss, causing communication difficulties (Global Burden of Disease 2019 Ageing Collaborators 2022). These health‐related challenges and conditions can greatly affect quality of life, ability to perform daily activities, engage in social activities, and lead to social isolation and communication difficulties.

It is important to identify the proportion of the ageing population that will require aged‐care services and supports because of health‐related challenges and conditions. The available data internationally that compares use across different age categories is limited for reporting standardised data to enable valid international comparisons (Jain et al. 2019). The proportion of older Australians aged ≥ 65 years who use aged care services remains consistent at just over 5% over the last decade (Khadka et al. 2019), and those requiring permanent residential long‐term care are 10% aged 65–74 years, 30% aged 75–84 years and 58% ≥ 85 years (compared with 41% in home care and 30% in home support) (Australian Institute of Health and Welfare (AIHW) 2024; Gibson 2020). In countries like Korea and Japan, it is < 10% of the population aged 80 years and older and < 5% for those 65 years and older. However, the care and support needs of older adults are consistent across regions to include a range of physical, social and psychological challenges due to living with chronic conditions. The care and support needs of older adults are non‐medical (The Lancet Healthy 2021), and a recent review categorised these to include social activities and relationships, psychological health, and activities related to mobility, self‐care, and domestic life (Abdi et al. 2019). This review also highlights older adults have a desire to cope with their illness and maintain independence; however, environmental factors interfered with these efforts because of a lack of professional advice on self‐care strategies, poor communication and coordination of services, and a lack of information on services such as care pathways.

Distinguishing between primary and secondary ageing may be important for understanding why all older adults are not susceptible or at risk for chronic conditions or may not require long‐term ageing care. Primary ageing refers to the gradual age‐related changes over which we have no control, reflecting the biological aspects of ageing that affect everyone, whereas secondary ageing is more individual and is influenced by health behaviours, lifestyle, and environmental factors such as lack of exercise, smoking, alcohol consumption, obesity and disease. Getting older will involve primary ageing for a higher proportion of the ageing population and therefore, many older adults may not experience the health‐related challenges associated with secondary ageing. The focus of health and social care approaches and interventions should be to maintain good health in all older adults in the population 65 years and older for as long as possible, with a strong focus on secondary ageing prevention in those that may be at risk. Secondary ageing prevention strategies will target those diseases that have the greatest impact on the health and wellbeing of older adults, to include cardiovascular and cerebrovascular disease, globally the two biggest causes of mortality. Addressing the health‐related challenges of ageing requires a multifaceted approach involving healthcare providers, carers, and older adults themselves. By focusing on prevention, early intervention, and comprehensive care, it is possible to improve the quality of life and health outcomes for older adults. Some regions in the world exemplify this and have been identified as Blue Zones (e.g., Loma Linda, CA, USA; Nicoya, Costa Rica; Sardinia, Italy; Ikaria, Greece; Okinawa, Japan) where older adults live a longer and healthier life have been identified as Blue Zones (Buettner and Skemp 2016). Older populations in these regions succeeded in maintaining a traditional lifestyle implying an intense physical activity that extends beyond the age of 80 years, a reduced level of stress, and intensive family and community support and consumption of locally produced foods (Buettner and Skemp 2016). The Blue Zones project is a good example for health and social care stakeholders and policymakers to ensure all aspects of communities and environments are designed to ensure the healthiest lifestyles possible for older adults to make healthy choices the easy and unavoidable choices.

## 4 Approaches Towards Population Ageing

Some of the concerns for addressing population ageing are the ability of families and communities, as well as modern social, political, economic, and health service delivery systems, to provide optimal support to older adults for improved health and quality of life. Additionally, health, social, and economic policies for older persons vary substantially across different countries. There is a dire need for effective policies that are inclusive of a systems' thinking and person‐centred approach at the macro level and an integrated, interdisciplinary, intergenerational, involvement of stakeholders (older adults' involvement in their own decision‐making) at the micro level for enhancing the health status, as well as the social and economic well‐being, of older populations. It is important for health professionals involved in the health and social care of older adults to understand what constitutes healthy and non‐healthy ageing. Ensuring all health professionals have sufficient training and education on the broader principles of gerontology and gerontological science may lead to less ageist‐directed decisions made at individual, community, and population levels when referring to certain chronological age groups. Greater encouragement for health and social care providers to lean towards the use of frameworks and models of care that apply the concept of healthy or active ageing in clinical settings is required.

### 4.1 Frameworks and Models of Health and Social Care for Use in Older Populations

Models of care for older adults require greater focus on health maintenance and sustainability that have a holistic human health approach addressing physical, psychological, social and environmental wellbeing and satisfaction. Pathology‐focused or disease‐based models work well in the context of multimorbidity, but these models often imply all older adults may be associated with an array of co‐morbidities and not represent those with no existing disease or conditions. Some theoretical frameworks and models have highlighted and outlined how healthy ageing individuals adapt to everyday life situations. Lifespan developmental approaches have made great efforts in bringing attention to a healthier approach towards ageing, and these approaches are considered the science of describing and explaining constancy and changes in human behaviour throughout the life course. They are also referred to as ontogenesis, defined as the development of an individual organism from birth to death (Baltes 1987; Li and Freund 2005). Baltes (1987) proposes that ontogenetic development is a life‐long process where no age period holds supremacy in regulating the nature of development during which all stages of the lifespan, both continuous and discontinuous processes, are at work (Baltes 1987). Developmental change involves gains and losses in different functional and life domains, and efforts to keep this balance favourable represent an essential aspect of human action and the momentum of personal development over the life span (Brandtstadter and Renner 1990). The developmental dynamic of positive (gains) and negative (losses) change led to life span work on specifying a general process of adaptation that would represent a lifelong dynamic relation between gain and loss that has been outlined in a theoretical framework describing successful ageing known as the Selection, Optimisation and Compensation (SOC) model (Baltes and Baltes 1990). Other lifespan theories of development include the Life‐span Theory of Control (Heckhausen and Schulz 1995) and assimilative and accommodative coping (Brandtstadter and Renner 1990). The SOC model is considered one of the leading models of lifespan development because it describes the processes of successful ageing instead of solely defining the end points (Ouwehand, de Ridder, and Bensing 2007). Lifespan developmental approaches were formulated to be highly general; hence, they have been considered as meta‐theories of development with great significance for application in the context of adaptation to the gains and losses that accompany older adult years of development (Donnellan and O'Neill 2014). In the context of gerontological care management, the use of theories or theoretical frameworks for guidance or to establish guidelines on how to care for older adults and how to support them in adapting to ageing processes remains limited.

## 5 Implications for Practice, Policy, and Research

At a systems or organisational level, there are significant economic implications if health and social care stakeholders and policy makers do not consider reverting to using a healthy ageing approach over continuing to use the conventional aged care approach for all older adults when they reach a certain chronological age, e.g., 65 years of age and over. Health professionals involved in the provision of health and social care for older adults may need to reimagine their perspectives on ageing through greater interprofessional research and systemic and service changes across disciplines (Jeyasingam et al. 2023) and use efforts that translate evidence‐based research on healthy ageing into clinical practice. In the full cycle of life, we also need to consider palliative care in the future for older adults now living well and healthy into their later years and that they too will reach the end of life, meaning healthy ageing will not make it possible for human beings to live forever.

We are at a nexus in health and social care provision to implicitly challenge the current medicalised approach used for enhancing older adults' living and available social programs and policies generated. There is a need for greater support for research innovation in technologies and services to facilitate an ageing population and improvement in therapies and treatments to keep older adults healthy in later life, remaining independent and active for as long as possible. A coordinated research investigation of systems that control organisation of care at local, national, and international levels and the decision‐making processes of health professionals involved in the management and care of older adults with age‐related health challenges is warranted. An audit and feedback model are required to investigate quality and performance enhancement regarding health systems of care for monitoring and managing older adults that are more challenged with age‐related conditions. Healthy ageing is a relatively recent field of interest in gerontological care for idenitfying the challenges it poses for the design of health and social care policy and practice. Promoting healthy ageing and recognising ageing indicators that are realistic for identifying functional and intrinsic capacity associated with ageing will be very supportive in response to the demands of a growing ageing population. Shifting health professionals' mindset in relation to ageing from a pathology‐orientated medical model to using a holistic healthy ageing framework will also support older adults' maintenance of high physical and cognitive function and sustained engagement in social and productive activities.

## Conflicts of Interest

The author declares no conflicts of interest.

## Data Availability

Data sharing is not applicable to this article as no new data were created or analyzed in this study.
